# Leptin Stimulates Prolactin mRNA Expression in the Goldfish Pituitary through a Combination of the PI3K/Akt/mTOR, MKK_3/6_/p^38^MAPK and MEK_1/2_/ERK_1/2_ Signalling Pathways

**DOI:** 10.3390/ijms18122781

**Published:** 2017-12-20

**Authors:** Aifen Yan, Yanfeng Chen, Shuang Chen, Shuisheng Li, Yong Zhang, Jirong Jia, Hui Yu, Lian Liu, Fang Liu, Chaoqun Hu, Dongsheng Tang, Ting Chen

**Affiliations:** 1 Foshan University, Foshan 528000, China; yanaifen@mail3.sysu.edu.cn (A.Y.); chyfwf@hotmail.com (Y.C.); yu71hui@126.com (H.Y.); lian2004@163.com (L.L.); 1115fang@sina.com (F.L.); 2 The Beijing Genomics Institute (BGI), Shenzhen 518083, China; chenss@connect.hku.hk; 3 State Key Laboratory of Biocontrol, Institute of Aquatic Economic Animals, and the Guangdong Province Key Laboratory for Aquatic Economic Animals, School of Life Sciences, Sun Yat-Sen University, Guangzhou 510275, China; lshuish@mail.sysu.edu.cn (S.L.); lsszy@mail.sysu.edu.cn (Y.Z.); jiajr@mail2.sysu.edu.cn (J.J.); 4 CAS Key Laboratory of Tropical Marine Bio-resources and Ecology (LMB), Guangdong Provincial Key Laboratory of Applied Marine Biology (LAMB), South China Sea Institute of Oceanology, Chinese Academy of Sciences, Guangzhou 510301, China; hucq@scsio.ac.cn

**Keywords:** goldfish, leptin, prolactin, pituitary, expression regulation, signal pathway

## Abstract

Leptin actions at the pituitary level have been extensively investigated in mammalian species, but remain insufficiently characterized in lower vertebrates, especially in teleost fish. Prolactin (PRL) is a pituitary hormone of central importance to osmoregulation in fish. Using goldfish as a model, we examined the global and brain-pituitary distribution of a leptin receptor (lepR) and examined the relationship between expression of *lepR* and major pituitary hormones in different pituitary regions. The effects of recombinant goldfish leptin-AI and leptin-AII on *PRL* mRNA expression in the pituitary were further analysed, and the mechanisms underlying signal transduction for leptin-induced *PRL* expression were determined by pharmacological approaches. Our results showed that goldfish *lepR* is abundantly expressed in the brain-pituitary regions, with highly overlapping *PRL* transcripts within the pituitary. Recombinant goldfish leptin-AI and leptin-AII proteins could stimulate *PRL* mRNA expression in dose- and time-dependent manners in the goldfish pituitary, by both intraperitoneal injection and primary cell incubation approaches. Moreover, the PI3K/Akt/mTOR, MKK_3/6_/p^38^MAPK, and MEK_1/2_/ERK_1/2_—but not JAK2/STAT 1, 3 and 5 cascades—were involved in leptin-induced *PRL* mRNA expression in the goldfish pituitary.

## 1. Introduction

Leptin is the protein product of the *obese* gene, and was first identified in mouse adipose tissue by positional cloning in 1994 [1]. Mammalian leptin is primarily secreted by white adipose tissue, and was initially identified as an anti-obesity hormone [2,3,4]. Additional studies have shown increasing evidence suggesting that leptin is a multifunctional hormone that plays other roles in the regulation of reproduction [5], metabolism [6], immunity [7], and pituitary hormone synthesis and secretion [8].

Fish leptin was first identified in *Fugu rubripes* by gene synteny analysis [9], followed by similar discoveries in other teleost species, such as common carp [10], medaka [11], zebrafish [12], salmon [13], tilapia [14,15], and minnow [16]. The fish *leptin* mRNA is abundantly expressed in the liver, but is lacking in the adipose tissue [17,18]. Leptin signalling in fish possibly serves as an integrating system that includes energy metabolism, reproduction, and stress [17,18]. However, the role of leptin as a satiety factor in fish still remains controversial, and is not as dogmatically defined as its counterparts in mammals [18].

The actions of leptin are mediated by its receptor, namely, leptin receptor (lepR). LepR is a single-transmembrane receptor encoded by the *diabetes* (*db*) gene. Structurally, lepR can be divided into three parts: extracellular domain, transmembrane domain, and cytoplasmic domain. To date, six isoforms of lepR have been observed in humans, including a long-form receptor (lepRb), four short-form receptors (lepRa, lepRc, lepRd and lepRf), and a soluble-form receptor (lepRe) [19]. All of these receptors share an identical extracellular domain, but differ by the length of their transmembrane domain and cytoplasmic domain. Only lepRb has an intact intracellular domain that contains two Janus kinase 2 (JAK2) boxes and a signal transducer and activator of transcription (STAT) box [20]. The JAK/STAT pathway is the most illuminated intracellular signalling pathway for leptin actions. Upon ligand binding, JAK2 is phosphorylated, and STAT3 is subsequently activated. The activation of the JAK/STAT pathway by leptin administration is present in both wild-type and *ob*/*ob* mice, but not in *db*/*db* mice [21]. Other STAT transcription factors, such as STAT1, STAT5 and STAT6, are also involved in the lepRb-mediated downstream signalling of leptin [22]. In addition, studies have shown that the phosphatidylinositide 3-kinases (PI3K)/protein kinase B (Akt)/mechanistic target of rapamycin (mTOR) pathway is involved in the leptin action regulating food intake [23]. In specific target cells, leptin can activate mitogen-activated protein kinase (MAPK)-dependent signal pathways, including p^38^MAPK (MKK_3/6_ and p^38^MAPK) and p^42/44^MAPK [(MEK_1/2_ and extracellular signal–regulated kinases (ERK_1/2_)] cascades [24]. The cDNA sequences of *lepR* that correspond to human *lepR*b have been cloned in 19 species of teleost fish [16,25], but the signal transduction mechanisms for the leptin/lepR system in the fish models are not well-defined to date.

Leptin exerts its appetite-inhibiting effects by acting on the appetite control centres of the brain, particularly those within the hypothalamus. Leptin regulates a group of hypothalamic neuropeptides, and subsequently induces the response of pituitary hormone production and secretion via the hypothalamus-pituitary axis [26]. Based on evidence that *lepR* mRNAs are widely found in different types of pituitary cells [27,28,29], studies suggest that leptin may directly regulate the expression and secretion of pituitary hormones in the pituitary, by the activation of the leptin receptor [8]. In mammalian studies, leptin has been extensively scrutinised for its neuroendocrine functions, directly at the pituitary level or indirectly through hypothalamic mediation, such as promoting the secretion of growth hormone (GH), prolactin (PRL), thyroid stimulating hormone (TSH), gonadotropin hormones ((GTH), including follicle-stimulating hormone (FSH) and luteinizing hormone (LH)), as well as inhibiting the secretion of adrenocorticotropic hormone (ACTH) [30]. However, leptin actions in the regulation of pituitary hormones in fish models have not been fully elucidated. In tilapia, recombinant tilapia leptin-A suppressed pituitary GH accumulation and *GH* mRNA expression, but not GH release by in vitro incubation [31]. In European sea bass, the incubation of mouse leptin caused a significant increase of LH [32] and somatolactin (SL) [33] release in pituitary primary cells. In rainbow trout, human leptin treatment induced FSH release in pituitary cells only when the fish were sexually matured [34]. In bighead carp pituitary cells, an in vitro study showed that mammalian leptin could stimulate the expression of *TSH* mRNA [35]. However, mammalian leptin was used as the test substrate in most of experiments mentioned above, except for those performed in tilapia [31], and the mammalian leptins (from human or mouse) share less than 20% amino acid sequence homology with the fish leptins. Moreover, the binding of mammalian leptins to fish lepR was considerably lower than that of fish leptins [15].

Prolactin is an important pituitary hormone that promotes lactation in mammals, whereas in fish, PRL plays an important role in freshwater osmoregulation [36]. The importance of PRL highlights the need for defining the molecular determinants and intracellular mechanisms that regulate its synthesis and secretion from the pituitary. The injection of leptin could elevate circulating PRL levels in rats [37]. Leptin may also stimulate PRL secretion in bovine pituitary explants [38], but not in primary cultures of the porcine anterior pituitary [39]. In tilapia, intraperitoneal (IP) injection of low-dosage recombinant tilapia leptin-A (0.5 μg/g body weight (bwt)) significantly stimulated pituitary PRL mRNA expression, while a high dosage of leptin-A (5 μg/g bwt) caused a significant reduction of pituitary PRL at the transcription level [40]. Moreover, recombinant human leptin potently increased the release of tilapia PRL via the ERK_1/2_ signal pathway during in vitro incubation [41]. However, two other important cascades for leptin signalling, including JAK/STAT and PI3K/Akt/mTOR pathways, have not been investigated to date in leptin-regulated PRL expression in teleost fish.

Goldfish (*Carassiusauratus*) are a freshwater fish belonging to the family Cyprinidae, under the order Cypriniformes. Goldfish are a well-established model for the regulation of pituitary hormone production and secretion [42]. In goldfish, two *leptin* genes (GenBank: FJ534535.1 and FJ854572.1) have been reported as *leptin*-AI and *leptin*-AII [43,44]. Previous studies have shown that the administration of recombinant mouse leptin may reduce food consumption [45,46], body weight [45], and locomotor activity of goldfish [46]. The effects of mouse leptin on goldfish feeding could be blocked by neuropeptide Y (NPY) or orexin [47], but reinforced by cocaine-amphetamine-regulated transcript (CART) and cholecystokinin (CCK) [47]. The recombinant goldfish *leptin*-AI and *leptin*-AII have been generated in our lab by the methylotrophic yeast expression system, and have shown effects on the inhibition of feeding behaviours and the reduction of food consumption, via a mediation of specific central appetite regulators [43]. The mechanisms for glucagon-regulated goldfish *leptin*-AI and *leptin*-AII expression have also been investigated [48]. To further develop the pituitary actions of leptin in goldfish, in the present study, the tissue and brain-pituitary expression patterns of *lepR* in goldfish were examined, and the expression profiles of *leptin*s, *lepR* and other major hormones in different parts of the pituitary were characterized. The in vivo and in vitro effects of goldfish recombinant leptins on *PRL* mRNA expression, and the involved signal transduction mechanisms, were also investigated. To the best of our knowledge, the present study is the first to examine PRL regulation by leptin in a Cyprinidae fish, and it may reveal the potential roles of leptin in osmoregulation.

## 2. Results

### 2.1. In Vivo and In Vitro Regulation of PRL Transcript by Leptin

**Figure 1:**
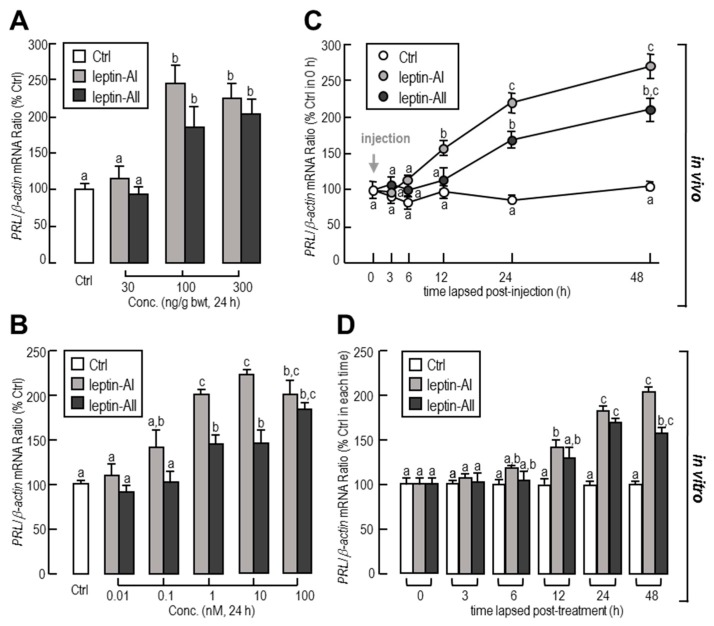
In vivo and in vitro effects of leptin-AI and leptin-AII treatment on *PRL* transcripts in goldfish. Dose- (**A**) and time-dependent (**B**) effects of leptin-AI or leptin-AII IP injection on *PRL* mRNA expression in the pituitary. For time-course experiments, goldfish were IP injected with leptin-AI or leptin-AII at 100 ng/g for 0, 3, 6, 12, 24 and 48 h. For dose-dependence experiments, the goldfish were IP-injected with leptin-AI or leptin-AII for 24 h with increasing doses (1–100 ng/g). Dose- (**C**) and time-dependent (**D**) effects of leptin-AI or leptin-AII incubation on *PRL* mRNA expression in primary cultured pituitary cells. For time-course experiments, goldfish pituitary cells were incubated for 3, 6, 12, 24, and 48 h with leptin-AI or leptin-AII, at a concentration of 100 nM. For dose-dependence experiments, goldfish pituitary cells were incubated with leptin-AI or leptin-AII for 24 h with increasing doses (0.01–100 nM). In these studies, data are expressed as the mean ± standard error (SE, *n* = 10 for the in vivo study, and *n* = 4 for the in vitro study). The same letter represents a similar level of transcriptional expression (*p* > 0.05), and a different letter represents significant difference in levels of transcriptional expression between two groups (*p* < 0.05).

To investigate the effects of leptin on *PRL* gene expression in the goldfish pituitary, the approach of IP injection was used. In this case, leptin mediated a time-dependent increase in pituitary *PRL* transcript expression. The maximal stimulatory responses for *PRL* mRNA to leptin-AI and leptin-AII administration were both observed at 48 h (Figure 1A). In dosage experiments, both leptin-AI and leptin-AII injection at concentrations of 100 and 300 ng/g, but not 30 ng/g, induced a significant increase in *PRL* mRNA levels (Figure 1A). The maximal stimulatory responses for *PRL* mRNA to leptin-AI and leptin-AII administration occurred at 48 h (Figure 1B).

To further confirm whether the regulation of *PRL* expression was directly affected by leptin, leptin was routinely added to goldfish pituitary primary cells for static incubation. In in vitro primary cell culture, increasing concentrations of leptins could elevate the transcriptional levels of *PRL* in dose-dependent manners (Figure 1C). The maximal responses to leptin-AI and leptin-AII on *PRL* transcripts were observed with treatment dosages of 10 and 100 nM, respectively. Additionally, the basal levels of *PRL* mRNA were stimulated by leptin administration in dose-dependent manners (Figure 1D). The maximal effects of leptin-AI and leptin-AII were observed at 48 and 24 h after treatment, respectively.

### 2.2. Tissue Expression Profiles of lepR and Expression Profiles of Major Hormones in Different Parts of the Pituitary

**Figure 2:**
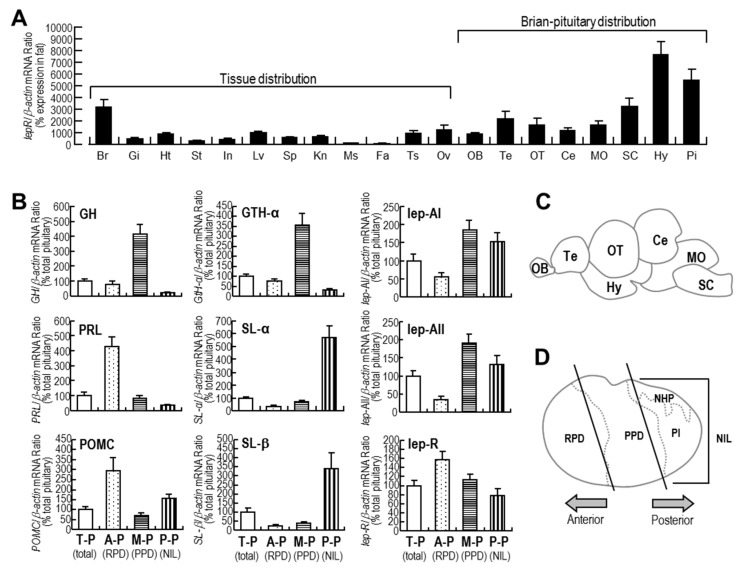
(**A**) Expression profiles of goldfish *lepR* in various tissues and brain regions, including brain (Br), gill (Gi), heart (Ht), intestine (In), liver (Lv), spleen (Sp), kidney (Kn), muscle (Ms), Fat (Fa), testis (Ts), ovary (Ov), the olfactory bulb (OB), telencephalon (Te), optic tectum (OT), cerebellum (Ce), medulla oblongata (MO), spinal cord (SC), hypothalamus (Hy), and pituitary (Pi), as assessed by real-time quantitative (qPCR); (**B**) Expression pattern of major hormones, including *GH*, *PRL*, *POMC*, *GTH-α*, *SL-α*, *SL-β*, *leptin*-AI, *leptin*-AII and *lepR,* in different regions of pituitary assessed by qPCR. T-P indicates total pituitary; A-P indicates anterior pituitary, corresponding to rostral pars distalis (RPD) in mammalian pituitary; M-P indicates medium pituitary, corresponding to proximal pars distalis (PPD) in mammalian pituitary; P-P indicates posterior pituitary corresponding to neurointermediate lobe (NIL), consisting of neurohypophysis (NHP) and pars intermedia (PI) in mammalian pituitary; (**C**) diagram showing the goldfish brain regions; (**D**) diagram showing different parts of the goldfish pituitary. In these studies, data are expressed as the mean ± SE (*n* = 3 for testis and ovary, and *n* = 6 for other tissue and pituitary samples).

As shown in Figure 2A, *lepR* mRNA was widely distributed in the goldfish central nervous system and peripheral tissues, with high expression levels in the brain, pituitary, heart, liver, testis, and ovary. Additionally, *lepR* mRNA was detected in all of the brain regions tested with the highest expression level in the hypothalamus.

As shown in Figure 2B, *GH* and *GTH-α* mRNAs were mainly expressed in the medium pituitary. *PRL* and proopiomelanocortin (*POMC)* mRNA expression was mainly detected in the anterior pituitary. *SL-α* and *SL-β* mRNA expression was mainly distributed in the posterior pituitary, and *leptin*-AI and *leptin*-AII mRNA was mainly expressed in the medium pituitary and posterior pituitary. However, *lepR* mRNA was evenly distributed in the anterior pituitary, medium pituitary, and posterior pituitary, with the highest expression level in the anterior pituitary, indicating that the expression profile of *lepR* highly overlapped with that of *PRL* within the goldfish pituitary.

### 2.3. Signal Pathways Involved in Pituitary PRL mRNA Expression Regulated by Leptin

**Figure 3:**
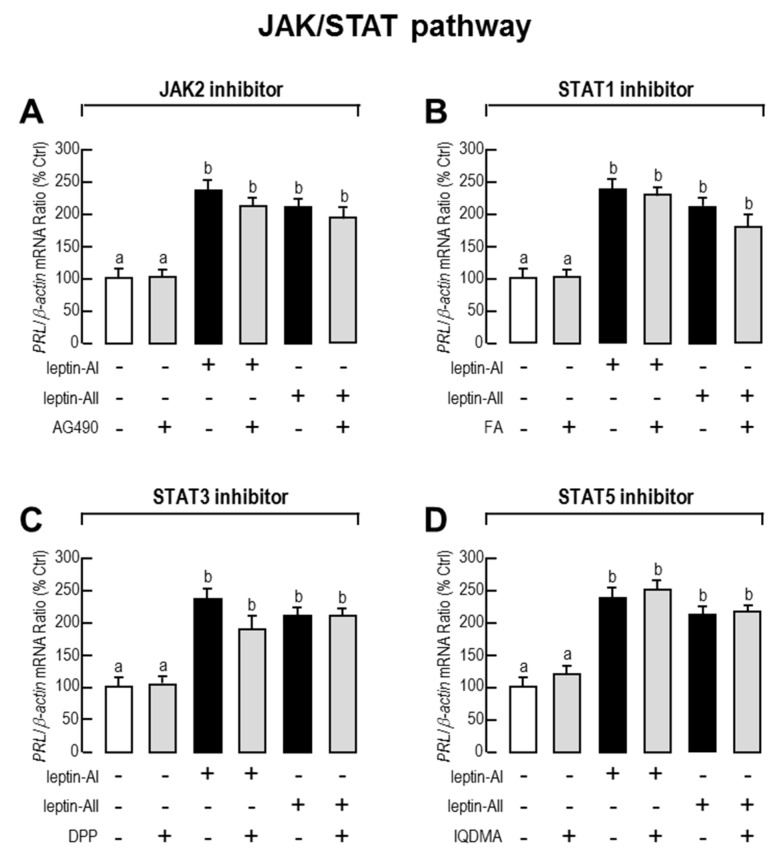
Effects of JAK/STAT signal pathway blockers on leptin-stimulated *PRL* mRNA levels in primary cultured goldfish pituitary cells. Goldfish pituitary cells were treated with leptin-AI (100 nM) or leptin-AII (100 nM), in the presence or absence of JAK2 inhibitor AG490 (50 μM, **A**), STAT1 inhibitor FA (10 μM, **B**), STAT3 inhibitor DPP (500 nM, **C**), and STAT5 inhibitor IQDMA (20 μM, **D**) for 24 h. In the present study, the data are expressed as mean ± SE (*n* = 4). The same letter represents a similar level of transcriptional expression (*p* > 0.05), and a different letter represents significant difference in levels of transcriptional expression between two groups (*p* < 0.05).

**Figure 4:**
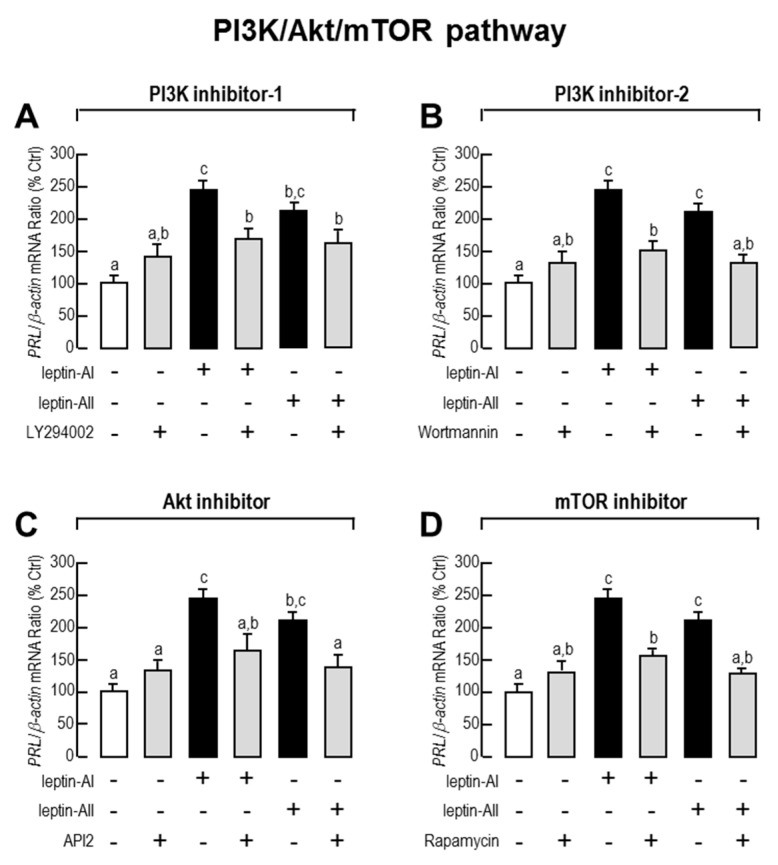
Effects of PI3K/Akt/mTOR signal pathway blockers on leptin-stimulated *PRL* mRNA levels in primary cultured goldfish pituitary cells. Goldfish pituitary cells were treated with leptin-AI (100 nM) or leptin-AII (100 nM) in the presence or absence of PI3K inhibitors LY294002 (10 μM, **A**) and wortmannin (100 nM, **B**), Akt inhibitor API2 (100 nM, **C**), and mTOR inhibitor rapamycin (20 nM, **D**) for 24 h. In the present study, the data are expressed as mean ± SE (*n* = 4). The same letter represents a similar level of transcriptional expression (*p* > 0.05), and a different letter represents significant difference in levels of transcriptional expression between two groups (*p* < 0.05).

**Figure 5:**
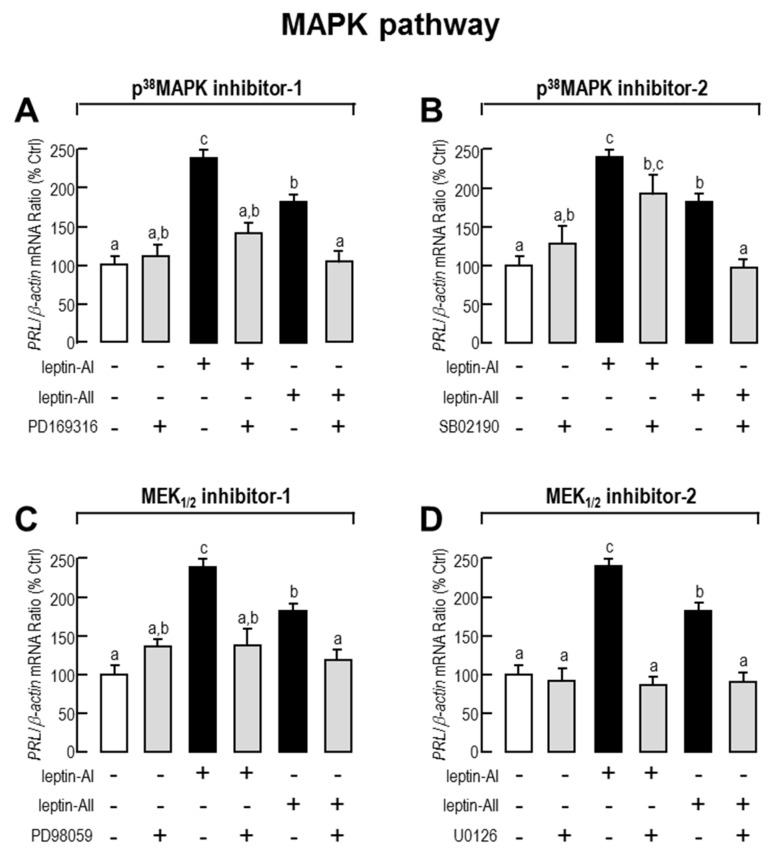
Effects of MAPK signal pathway blockers on leptin-stimulated *PRL* mRNA levels in primary cultured goldfish pituitary cells. Goldfish pituitary cells were treated with leptin-AI (100 nM) or leptin-AII (100 nM) in the presence or absence of p^38^MAPK inhibitors PD169316 (100 nM, **A**) and SB02190 (100 nM, **B**), MEK_1/2_ inhibitors PD98059 (10 μM, **C**) and U0126 (200 nM, **D**), for 24 h. In the present study, the data are expressed as mean ± SE (*n* = 4). The same letter represents a similar level of transcriptional expression (*p* > 0.05), and a different letter represents significant difference in levels of transcriptional expression between the two groups (*p* < 0.05).

Given that JAK/STAT (STAT-1, STAT-3 and STAT-5), PI3K/Akt/mTOR and MAPK (p^38^MAPK and p^42/44^MAPK) cascades are the major intracellular signal pathways activated by leptin, we used the corresponding pathway inhibitors to understand the roles of these pathways in leptin-mediated *PRL* mRNA expression. As shown in Figure 3, Figure 4 and Figure 5, the expression levels of *PRL* mRNA were significantly stimulated by leptin-AI or leptin-AII, in the absence of signalling inhibitors. For the JAK/STAT cascades, as shown in Figure 3, the leptin-induced *PRL* mRNA expression could not be blocked by any inhibitors for JAK/STAT cascades, including the JAK2 inhibitor AG490 (50 μM), the STAT1 inhibitor fludarabine (FA, 10 μM), the STAT3 inhibitor 5,15-Diphenyl-21H,23H-porphine (DPP, 500 nM) or the STAT5 inhibitor IQDMA (20 μM). In contrast, as shown in Figure 4, leptin-induced *PRL* mRNA expression could be totally abolished by co-treatment with the PI3K inhibitors LY294002 (10 μM) and wortmannin (100 nM), the Akt inhibitor API2 (100 nM) or the mTOR inhibitor rapamycin (20 nM). Furthermore, as shown in Figure 5, leptin-induced *PRL* mRNA expression could be totally abolished by co-treatment with the p^38^MAPK inhibitors PD169316 (100 nM), or the MEK_1/2_ inhibitors PD98059 (10 μM) and U0126 (200nM). However, the p38MAPK inhibitor SB02190 (100 nM) could only block the leptin-AI-, but not the leptin-AII-induced *PRL* expression. Taken as a whole, these results indicate that the PI3K/Akt/mTOR, MKK_3/6_/p^38^MAPK, and MEK_1/2_/ERK_1/2_ cascades were involved in the regulation of *PRL* mRNA expression by leptin in the goldfish pituitary.

## 3. Discussion

In teleost fish, PRL has been characterized as a pleiotropic pituitary hormone with diverse functions [49], with the assumption that its best-known physiological role is to serve as a regulator of salinity homoeostasis and ion transport for freshwater adaptation [50,51]. Additionally, a study in tilapia showed that the hepatic *leptin*-A and *lepR* mRNA levels might increase during acute hyperosmotic stress, implying a potential role for leptin in osmoregulation in fish [14]. The present study provided evidence for the leptin-mediated induction of *PRL* mRNA at the pituitary level in a freshwater Cyprinidae fish, suggesting a link between these two osmoregulatory hormones, respectively produced in the liver and the pituitary. Considering that PRL may inhibit the hepatic *leptin* mRNA expression in fish, a negative feedback regulatory model of these two hormones has also been proposed [40]. In common carp, recombinant human leptin attenuates the stress axis at multiple levels [52]. The role of leptin in osmoregulation has been exhibited in salmon [17] and tilapia [40] by its regulatory effects on PRL. However, more evidence to confirm the leptin function in osmoregulation should be developed by studying its effects on the survival of fish under hyperosmotic or hypoosmotic conditions.

In mammals, certain neuroendocrine factors in the hypothalamus, such as thyrotropin-releasing hormone (TRH), prolactin-releasing peptide (PRP), pituitary adenylate cyclase-activating peptide (PACAP) and prolactin-releasing peptide (PRP), stimulate PRL secretion in the anterior pituitary, while other factors, such as dopamine (DA) and somatostatin (SS), inhibit the secretion of PRL [53]. In teleost fish, the production and/or secretion of PRL could be enhanced by gonadotropin-releasing hormone (GnRH), ghrelin, PACAP, estradiol (E_2_), testosterone (T), TRH, GH, LH, insulin-like growth factor (IGF), and PACAP, and reduced by DA, cortisol, somatostatin (SS), urotensin-II (U-II), and vasoactive intestinal peptide (VIP) [49,54,55]. In addition, leptin has also been demonstrated to be a stimulator for pituitary PRL release in mice [37] and bovine [38]. In an in vitro experiment on the tilapia pituitary, incubation with a low dosage (1–10 nM) of recombinant human leptin induced the secretion of PRL [41]. By using an in vivo IP injection approach, the *PRL* mRNA in the tilapia pituitary increased after a low dosage (0.5 μg/g bwt), but decreased after a high dosage (5 μg/g bwt) of tilapia leptin-A administration [40]. The results of the present study showed that the minimum dosages for goldfish leptin-AI- or Leptin-AII-induced *PRL* mRNA expression were 100 ng/g bwt (Figure 1A) and 1 nM (Figure 1C) in in vivo injection and in vitro incubation experiments, respectively, suggesting that the effects of leptin on pituitary *PRL* gene expression are similar, but not totally identical in different fish species. The plasma leptin levels of goldfish and other cyprinid fish are still unclear, but the plasma leptin level of tilapia is reported to range from 0.8 to 3.9 nM [40], indicating that leptin can affect *PRL* transcript at a physiological level. Additionally, the present study has shown that the effects of goldfish leptin-AI and leptin-AII on *PRL* gene expression were highly comparable, consistent with another report by our lab showing that the anorexigenic effects of these two leptin homologues were highly similar [43].

Although the liver is the main organ for leptin production in fish [17], goldfish *leptin*-AI and *leptin*-AII mRNAs are widely expressed in the central nervous system and peripheral tissues, including the pituitary [44]. In the present study, goldfish *lepR* mRNA was ubiquitously expressed, with high levels being detected in the hypothalamus and pituitary (Figure 2A), consistent with previous reports on *lepR* in other fish species [11,56,57,58,59].The detection of *leptin* and *lepR* mRNAs in the goldfish pituitary suggests that leptin might occur as an autocrine/paracrine factor, in parallel with its function as an endocrine factor derived from peripheral organs.

In mammals, PRL is synthesized in and secreted from specialized cells of the anterior pituitary gland, namely, the lactotrophs [53]. In humans, *leptin* is expressed in 20–25% of the anterior pituitary cells, which include 70%, 21%, 33%, 29%, 64% and 32% of ACTH, GH, FSH, LH and TSH cells, respectively, but is rarely colocalized with PRL cells (3%) [29]. In rats and mice, *leptin* is only expressed in a few pituitary cells (5–7%) and mainly colocalized with TSH cells (24-31%), while *lepR* is widely found in the normal pituitary and pituitary cell lines, including FS, GH3 and aT3-1 cells [27]. In the present study, both *PRL* and *lepR* mRNA expression was observed, with the highest expression levels in the anterior region of the pituitary in goldfish (Figure 2B), indicating that leptin could directly regulate *PRL* expression via activating the leptin receptor. However, indirect effects, such as those mediated by pituitary GH and/or LH via a paracrine manner [54], cannot be excluded.

**Figure 6:**
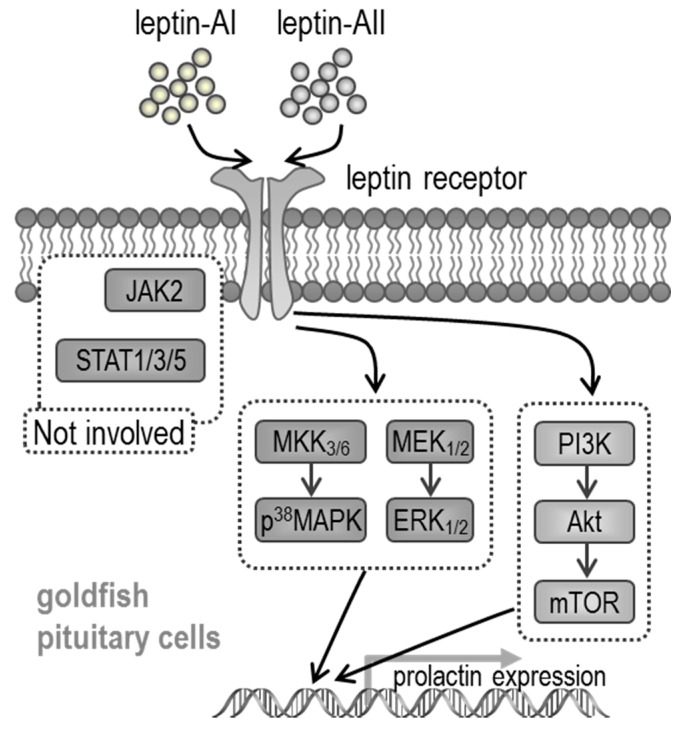
Working model for signal transduction mechanisms involved in leptin stimulation of *PRL* gene expression via lepR in the goldfish pituitary.

Based on the identification of *lepR* cDNA in a variety of fish species [11,15,56,57,59,60], the intracellular signal transduction mechanisms of fish leptin have also been developed. In rainbow trout, leptin incubation may trigger the phosphorylation of JAK2 and STAT3 in lepR-over-expressed RTH-149 cells [58], whereas the leptin-mediated acute anorexigenic effect is mediated by the hypothalamic PI3K-dependent pathway [61], and leptin-induced superoxide production is mediated by the STAT3 and NF-κB pathways and three major MAPK cascades [62]. In addition, the fish leptin affects lipid metabolism via the JAK2/STAT3 pathway in *Synechogobius hasta* [63], and enhances PRL release depending on ERK activation in tilapia [41]. However, the pituitary PRL synthesis and secretion is positively controlled by PACAP via cyclic adenosine monophosphate (cAMP)/protein kinase A (PKA) and Ca^2+^/CaM-dependent cascades in goldfish [55]. In the present study, we provide new information regarding leptin signalling for the regulation of pituitary hormones in a fish model. Given that the leptin-induced *PRL* gene expression could be blocked by the PI3K, Akt, mTOR, p^38^MAPK and MEK1/2 inhibitors in most cases, but did not respond to JAK2, STAT1, STAT3 or STAT5 inhibitors, it is speculated that this regulation is dependent on the coupling of PI3K/Akt/mTOR, MKK_3/6_/p^38^MAPK and MEK_1/2_/ERK_1/2_ signalling (Figure 6), which is more complicated than that previously described in tilapia [41]. However, some of the inhibitor experiments showed differently in specific cases; for example, the PI3K, Akt and mTOR inhibitors could only partially block the leptin effects, and the p^38^MAPK inhibitor SB02190 (100 nM) could only block the effects of leptin-AI, but not leptin-AII. The blockage capacity of the chemical inhibitors is dependent on their dosage, which is different between the mammalian and fish experiments.

In summary, by using goldfish as a model, we have demonstrated that leptin-AI and leptin-AII stimulated the expression of *PRL* mRNA in dose- and time-dependent manners in the pituitary, by both in vivo and in vitro approaches. We also demonstrated that the stimulatory effect was mediated through a functional coupling of PI3K/Akt/mTOR, MKK_3/6_/P^38^MAPK and MEK_1/2_/ERK_1/2_-dependent cascades. In the present working model (Figure 6), leptin-AI or leptin-AII activates lepR in the anterior pituitary. The PI3K/Akt/mTOR, MKK_3/6_/p^38^MAPK and MEK_1/2_/ERK_1/2_ signal pathways are subsequently activated, and finally induce the mRNA expression of the *PRL* gene. In contrast, the JAK/STAT cascades, including JAK2, STAT1, STAT3 and STAT5 elements in the JAK/STAT cascades, are not involved in the regulation of *PRL* expression by leptins. To the best of our knowledge, the present study provides the first evidence for leptin regulation of *PRL* gene expression in a Cyprinidae fish, thereby furnishing new insights into leptin-dependent osmoregulation and other leptin effects on pituitary hormones that warrant future investigation. Given that leptin is one of the predominant hormones for energy expenditure, our finding may build up an endocrine link between energy metabolism and osmoregulation.

## 4. Materials and Methods

### 4.1. Animals

Goldfish with body weights ranging from 25 to 30 g were purchased from the local aquarium market and maintained at 20–25 °C under a 12:12 h dark–light photoperiod, with a regular feeding schedule, for 14 days prior to the experiments. During the process of tissue sampling, the fish were anaesthetized by 0.05% tricainemethanesulfonate (MS222, Sigma, St. Louis, MO, USA), prior to being sacrificed by spinosectomy according to procedures approved by the Ethics Committees of Foshan University (FSYQ201417, 01-01-2015).

### 4.2. Test Substances

Goldfish leptin-AI and leptin-AII recombinant protein were previously expressed in yeast by our lab, and the protein activities were also tested [43]. For the pharmacological studies, AG490, FA, and DPP were purchased from Sigma, while IQDMA was purchased from Merck, and PD169316, SB203580, PD98059, U0126, LY294002, wortmannin, API-2 and rapamycin were purchased from Calbiochem. Test substances were prepared as 1 mM frozen stocks in small aliquots, and diluted with a pre-warmed culture medium to appropriate concentrations 15 min prior to drug treatment.

### 4.3. Tissue Distribution of lepR mRNA and Expression Profiles of lepR and Major Hormones in Different Regions of the Pituitary

The tissue distribution of *lepR* mRNA in goldfish was assessed by quantitative PCR (qPCR) in selected tissues. Three male and three female fish were used as individuals in this case, and the selected tissues included the brain, gill, heart, stomach, intestine, liver, spleen, kidney, muscle, fat, testis, and ovary. In addition, the distribution of *lepR* mRNA in the brain-pituitary regions, such as the olfactory bulb, telencephalon, optic tectum, cerebellum, medulla oblongata, spinal cord, hypothalamus, and pituitary, were also investigated.

Expression profiles of *GH*, *PRL*, *proopiomelanocortin* (*POMC*), *GTH-α*, *SL-α*, *SL-β*, *leptin*-AI, *leptin*-AII and *lepR* in different parts of the pituitary were examined by the qPCR technique. Goldfish pituitaries from the six individuals were each divided into three parts, including anterior pituitary (rostral pars distalis (RPD)), medium pituitary (proximal pars distalis (PPD)), and posterior pituitary (neurointermediate lobe (NIL), including pars intermedia (PI) and neurohypophysis (NHP)). The samples were frozen in liquid nitrogen and stored at −80 °C for RNA extraction and reverse transcription.

### 4.4. Intraperitoneal Injection and In Vivo Sample Collection

The in vivo effects of leptin-AI and leptin-AII on *PRL* mRNA expression in the goldfish pituitary were analysed with an IP injection approach, as previously described [48]. Briefly, after deep anaesthesia with 0.05% MS222, 100 μL of leptin solution at a final concentration of 300 ng/g bwt [43,47] dissolved in freshwater fish physiological saline (FFPS, [64]) was injected into the peritoneal cavity, using a 23-gauge needle attached to a 1 mL syringe. The injection of FFPS only was used as a control. The fish were routinely sacrificed at 0–48 h after injection for the time-course studies, or at 24 h for the dose-dependent studies. The pituitary samples were collected, frozen in liquid nitrogen, and stored at −80 °C for RNA extraction and reverse transcription.

### 4.5 Isolation, Primary Culture, and Static Incubation of Goldfish Pituitary Cells

The regulation of *PRL* transcript levels by leptin was further examined in goldfish pituitary primary cells. After anaesthesia with MS222, the goldfish were decapitated, then the pituitaries were removed and cut into 0.5 mm thick sections and digested with trypsin (2.5 mg/mL) at 28 °C for 30 minutes. Next, pituitary fragments of the digestion were suspended in a minimum essential medium (MEM) without Ca^2+^, and mechanically dispersed into single cells by gentle pipetting. The dispersed pituitary cells were then separated from the remaining fragments by filtration, through a sterile 30 μm mesh, and harvested by centrifugation at 1,000 × g for 10 min at 4 °C. The viability of the cells was assessed using a Trypan blue exclusion assay, and only preparations with more than 95% viability were used in subsequent experiments. The resulting pituitary cells were seeded onto a 24-well plate at a density of 2.5 × 10^6^ cells per well, in MEM containing 5% foetal bovine serum (FBS), then incubated at 25 °C overnight in 5% CO_2_ for recovery. On the second day after cell preparation, test substances prepared in MEM were gently overlaid onto pituitary cells, after the removal of the old culture medium. The cells were incubated with the test substances for another 3–48 h for time-course studies, or for 24 h for dose-dependent or pharmacological studies. Finally, the cells were harvested by dissolving them in TRIzol reagent (Invitrogen, Carlsbad, CA, USA).

### 4.6 Measurement of Transcriptional Expression of Target Genes by Quantitative PCR

Total RNA from the tested samples was isolated using TRIzol reagent, digested with DNase I (Invitrogen, Carlsbad, CA, USA) and reverse transcribed using PrimeScript™ RT kit (TaKaRa, Tokyo, Japan). Transcriptional expression of target genes (*PRL*, *leptin*-AI, *leptin*-AII, *lepR*, *GH*, *POMC*, *GTH-α*, *SL-α*, *SL-β*, and *β-actin*) was detected using SYBR Premix ExTaq™ II (TaKaRa, Tokyo, Japan), in a Rotor-GeneRG-3000 Real-time PCR System (Qiagen, Hilden, Germany), with primers and PCR conditions as previously reported (Table 1) [43,48,65]. Serially diluted plasmid DNAs containing ORF sequences for the target genes were used as the standards for qPCR. After PCR, the identities of the PCR products were routinely confirmed by melting curve analysis.

### 4.7. Data Transformation and Statistical Analysis

For qPCR, the raw data of target gene expression were expressed as fmol per tube, and routinely normalized as a ratio of *β-actin* mRNA detected in the same sample. Given that no significant differences were detected for *β-actin* expression in these experiments, the raw data of target gene expression were simply transformed as a percentage of the mean values of the control group for statistical analysis. Data were expressed as the mean ± standard error (mean ± SE), and analysed by using one-way ANOVA followed by Fisher’s least significant difference (LSD) test with SPSS (IBM Software). Differences were considered significant at *p* < 0.05.

## Reference

## Figures and Tables

**Table 1:** Primers and amplification conditions for the qPCR analysis in this study.

Gene Target/Accession No.(Primer Sequences, 5’–3’)	PCR Condition			Cycle
	Denaturing	Annealing	Extension	
***PRL*/ S82197**				
CACTCTCTCAGCACCTCTCTC	94 °C	58 °C	72 °C	× 35
CTCTTTGGTCTTGCTGTCAATG	30 s	30 s	30 s	
***lepR*/ EU911005**				
TCATCAACCCAAACGACG	94 °C	56 °C	72 °C	× 35
GTGAACTCCTCTGAGCCATA	30 s	30 s	30 s	
***leptin*-AI / FJ534535**				
TCCAAAGCTCCTCATAGG	94 °C	52 °C	72 °C	× 35
TGGTGGGTGGCGTTTTCC	30 s	30 s	30 s	
***leptin*-AII / FJ854572**				
TATCGTGGACACCCTAACTAC	94 °C	52 °C	72 °C	× 35
GGTCTAAAGCCAAGAACCCTAA	30 s	30 s	30 s	
***GH*/ EU157192**				
TTAACGACTTTGAGGACAGCCT	94 °C	58 °C	72 °C	× 35
CAGCTTCTCAGTGATCTGGTTG	30 s	30 s	30 s	
***GTH-α*/ AY800267**				
GCTCCTGTCTATCAGTGTATG	94 °C	58 °C	72 °C	× 35
GCACCCGTTTAACTTCTTT	30 s	30 s	30 s	
***SL-α*/ EU580712**				
ATATGTTTGTCCCGTACCCTCT	94 °C	56 °C	72 °C	× 35
TTTATCAGACACCCACTTGGTC	30 s	30 s	30 s	
***SL-*β/ CAU72940**				
AGGGACCATGTGTTCTCCTAAA	94 °C	56 °C	72 °C	× 35
AGAACCAGTATACCCTGCTCCA	30 s	30 s	30 s	
***POMC*/ AJ431209**				
AAGCGCTCCTACTCCATGGA	94 °C	60 °C	72 °C	× 35
CTCGTCCCAGGACTTCATGAA	30 s	30 s	30 s	
***β-actin*/ AB039726**				
CTGGTATCGTGATGGACTCT	94 °C	56 °C	72 °C	× 35
AGCTCATAGCTCTTCTCCAG	30 s	30 s	30 s	
